# Supplementary material for: Empirical methods for controlling false positives and estimating confidence in ChIP-Seq peaks
Source: BMC Bioinformatics. 2008 Dec 5;9:523. doi: 10.1186/1471-2105-9-523 (PMC2628906; doi:10.1186/1471-2105-9-523)
Supplement: Additional File 1 — A variety of html documents from the USeq web site detailing the available applications, their best usage, output file type descriptions, command line menus, etc. [file 1471-2105-9-523-S1.zip › USeqUserGuides/JavaDoc.zip!/JavaDoc/trans/main/IntervalFilter.html]

IntervalFilter


|  |  |  |
| --- | --- | --- |
| **Overview** \| **Package** \| **Class** \| **Use** \| **Tree** \| **Deprecated** \| **Index** \| **Help** | |  |
| **PREV CLASS**   **NEXT CLASS** | **FRAMES**    **NO FRAMES**     **All Classes** |  |
| SUMMARY: NESTED \| FIELD \| CONSTR \| METHOD | DETAIL: FIELD \| CONSTR \| METHOD |  |


---


## trans.main Class IntervalFilter

```
java.lang.Object
  trans.main.IntervalFilter
```

---

``` public class IntervalFilter extends java.lang.Object ```

Sorts an Interval[] into two groups, those that pass or fail a variety of filters.

---

**Constructor Summary**

`IntervalFilter(java.lang.String[] args)`


| **Method Summary** | |
| --- | --- |
| `void` | `deleteIntersectingIntervals()` |
| `void` | `deleteSpecificIntervals()` |
| `void` | `filterByStndDev()` |
| `void` | `filterSubWindow()`             Filters by the median ratio of the sub window |
| `void` | `filterWindowScore()` |
| `static void` | `main(java.lang.String[] args)` |
| `static void` | `printDocs()` |
| `void` | `processArgs(java.lang.String[] args)`             This method will process each argument and assign any new varibles |

**Methods inherited from class java.lang.Object**

`equals, getClass, hashCode, notify, notifyAll, toString, wait, wait, wait`

**Constructor Detail**

### IntervalFilter

```
public IntervalFilter(java.lang.String[] args)
```


**Method Detail**

### deleteSpecificIntervals

```
public void deleteSpecificIntervals()
```

---


### deleteIntersectingIntervals

```
public void deleteIntersectingIntervals()
```

---


### filterWindowScore

```
public void filterWindowScore()
```

---


### filterSubWindow

```
public void filterSubWindow()
```

:   Filters by the median ratio of the sub window

---


### filterByStndDev

```
public void filterByStndDev()
```

---


### printDocs

```
public static void printDocs()
```

---


### processArgs

```
public void processArgs(java.lang.String[] args)
```

:   This method will process each argument and assign any new varibles

---


### main

```
public static void main(java.lang.String[] args)
```


---


|  |  |  |
| --- | --- | --- |
| **Overview** \| **Package** \| **Class** \| **Use** \| **Tree** \| **Deprecated** \| **Index** \| **Help** | |  |
| **PREV CLASS**   **NEXT CLASS** | **FRAMES**    **NO FRAMES**     **All Classes** |  |
| SUMMARY: NESTED \| FIELD \| CONSTR \| METHOD | DETAIL: FIELD \| CONSTR \| METHOD |  |


---
